# Effectiveness and Perception of Flipped Classroom vs. Traditional Teaching Methods among Third Phase MBBS Students at a Medical College in Central India

**DOI:** 10.7759/cureus.103998

**Published:** 2026-02-20

**Authors:** Ashok B Najan, Sangeeta B Chinchole, Nazeem I Siddiqui

**Affiliations:** 1 Forensic Medicine and Toxicology, Nandkumar Singh Chouhan Government Medical College, Khandwa, IND; 2 Physiology, Nandkumar Singh Chouhan Government Medical College, Khandwa, IND

**Keywords:** competency-based medical education, flipped classroom, student perception, teaching–learning methods, active learning

## Abstract

Background: The flipped classroom model is an innovative teaching-learning approach that shifts content delivery outside the classroom and utilizes class time for active, student-centered learning. Evidence regarding its effectiveness in Indian undergraduate medical education remains limited.

Objectives: The objective of the study was to compare the effectiveness of the flipped classroom with traditional teaching methods and to assess students’ perceptions of the flipped classroom approach among third-phase MBBS students.

Methods: An educational interventional study was conducted among third-phase MBBS students using flipped classroom and traditional teaching methods. A total of 120 students participated in the study, generating 220 learning observations across two crossover teaching sessions. Knowledge gain was assessed using pre-test and post-test multiple-choice questionnaires. Students’ perceptions of the flipped classroom were evaluated using a validated five-point Likert scale questionnaire. Statistical analysis included paired and independent *t*-tests, with effect size calculated using Cohen’s *d*.

Results: Students exposed to the flipped classroom demonstrated significantly greater improvement in post-test scores compared to those taught using traditional methods, with a large effect size. Perception analysis revealed positive student attitudes toward the flipped classroom, particularly with respect to engagement, self-paced learning, and improved conceptual understanding.

Conclusion: The flipped classroom approach was more effective than traditional teaching methods in enhancing academic performance and was positively perceived by undergraduate medical students. Its integration into routine medical teaching may improve learning outcomes and support competency-based medical education.

## Introduction

Medical education is continuously evolving to meet the learning needs of students in the digital era, with increasing emphasis on learner-centered teaching-learning strategies rather than teacher-centered approaches [1]. Traditional lecture-based teaching, although effective for delivering information to large groups, often results in passive learning with limited student interaction and engagement [2].

Active learning techniques have been shown to promote deeper understanding, critical thinking, and long-term retention of knowledge, which are essential competencies for medical graduates [3]. The flipped classroom technique represents a pedagogical shift wherein students are exposed to core learning materials before classroom sessions, allowing face-to-face time to be utilized for interactive discussions, problem-solving, and application of concepts [1,4].

Many international studies have demonstrated that the flipped classroom approach improves student performance, engagement, and satisfaction in medical and health professions education [4-6]. In the Indian context, adoption of the flipped classroom remains limited, though early studies suggest it is both feasible and effective [7].

In competency-based medical education (CBME), introduced by the National Medical Commission, there is a strong need to incorporate teaching-learning methods that foster self-directed learning, integration, and higher-order cognitive skills [8]. This study was therefore undertaken to compare the effectiveness of flipped classroom teaching with traditional teaching methods and to assess students’ perceptions of the flipped classroom model among third-phase MBBS students in India.

## Materials and methods

This was a comparative interventional study conducted in the Department of Forensic Medicine and Toxicology, Nandkumar Singh Chouhan Government Medical College, Khandwa, Madhya Pradesh, India, over a period of three months from April 1, 2024, to June 30, 2024. The study used crossover teaching sessions, employed to minimize selection bias and ensure exposure of students to both teaching methods [9]. The study was approved by the Institutional Ethics Committee, Nandkumar Singh Chouhan Government Medical College (approval number: 141/IEC/NSCGMCK/2024).

Study participants

The batch of third-phase MBBS students (n=120) was divided into Batch A (roll numbers 1-60) and Batch B (roll numbers 61-120). Students who provided informed consent were included in the study. Students who failed to complete both pre-test and post-test assessments were excluded from the final analysis.

Teaching intervention

In the first teaching session, Batch A was designated as the flipped classroom group. Instructional materials, including recorded lectures, PowerPoint presentations, and reading notes, were shared with students prior to classroom sessions through online platforms. The scheduled one-hour classroom time was then utilized for higher-order learning activities such as application, analysis, and problem-solving through case-based discussions, group activities, quizzes, and concept clarification, thereby encouraging active participation and peer learning [4]. Batch B was designated as the traditional teaching group and received standard didactic lectures covering the same content in a conventional classroom setting, without prior distribution of learning materials [2].

In the subsequent teaching-learning session, a crossover of the batches was implemented. Batch A was assigned to the traditional teaching method, while Batch B was designated as the flipped classroom group.

Assessment of learning

Knowledge gain was assessed using multiple-choice question (MCQ)-based pre-intervention tests and post-intervention tests designed to evaluate students’ understanding of the taught topics (see Appendices). The questionnaire was administered through Google Forms (Google LLC, Mountain View, California, USA). For the two teaching sessions, four MCQ questionnaires were developed, each containing 10 questions. All questionnaires were prepared by a subject expert who was not involved in the teaching conducted during the study. The expert was instructed to construct MCQs with varying levels of difficulty, ranging from easy to difficult. The questionnaires were subsequently reviewed and validated by two senior subject experts to ensure content relevance and appropriateness. Different MCQ questionnaires with comparable levels of difficulty were used for pre-test and post-test assessments to minimize recall bias. This method of assessment has been widely used in educational research to measure short-term learning outcomes [6].

Perception questionnaire

Students’ perceptions of the flipped classroom were assessed using a validated five-point Likert scale questionnaire (1 = strongly disagree to 5 = strongly agree), focusing on engagement, conceptual clarity, self-directed learning, and overall satisfaction [7,10]. A total of seven open-ended questions were designed to record the perceptions.

Statistical analysis

Data were analyzed using appropriate statistical tests. Paired t-tests were used to compare pre-test and post-test scores within groups, while independent t-tests were used for between-group comparisons. Effect size was calculated using Cohen’s d to determine the magnitude of the educational impact. A p-value of less than 0.05 was considered statistically significant.

## Results

A total of 220 paired learning observations were obtained from 120 students across the two crossover teaching sessions, with each student contributing data from both teaching methods. Of these, 109 students were in the flipped classroom group (58 in Session 1 and 51 in Session 2), and 111 were in the traditional teaching group (54 in Session 1 and 57 in Session 2). Additionally, 108 students responded to the perception questionnaire. As shown in Table 1, both the flipped classroom and traditional teaching methods resulted in statistically significant improvements in post-test scores. However, the flipped classroom group demonstrated a significantly greater knowledge gain compared with the traditional teaching group (p < 0.0001). The effect size for the flipped classroom (Cohen’s d = 0.98) indicated a large educational impact, whereas the traditional teaching group showed a moderate effect size (Cohen’s d = 0.70). The mean knowledge gain was higher in the flipped classroom group (1.75) compared with the traditional teaching group (1.20). The between-group effect size was small-to-moderate (Cohen’s d = 0.34), favouring the flipped classroom. 

**Table 1:** Pre-test and Post-test Scores in Flipped Classroom and Traditional Teaching Groups Between-group effect size for knowledge gain: Cohen’s d = 0.34

Teaching Method	Pre-test, mean ± SD	Post-test, mean ± SD	Gain	p-value (within group)	Cohen’s d (within group)
Flipped Classroom	4.23 ± 1.54	5.98 ± 1.51	1.75	< 0.0001	0.98 (large effect)
Traditional Teaching	3.60 ± 1.47	4.80 ± 1.73	1.20	< 0.001	0.70 (moderate effect)

Analysis of the perception questionnaire revealed that a majority of students expressed positive attitudes toward the flipped classroom approach. As shown in Table 2, more than 80% of respondents agreed or strongly agreed that the flipped classroom improved engagement, conceptual clarity, and self-paced learning. Additionally, most students preferred the flipped classroom model for use in other subjects, indicating high acceptance and satisfaction.

**Table 2:** Student Perception of the Flipped Classroom Approach (N=108)

Statement	Agree/Strongly Agree, n (%)
Flipped classroom improved my understanding of the topic	89 (82.4%)
Flipped classroom increased my engagement during class	92 (85.2%)
Pre-class materials helped me learn at my own pace	95 (88.0%)
Classroom discussions clarified difficult concepts	90 (83.3%)
Flipped classroom encouraged active participation	87 (80.6%)
Flipped classroom should be used in other subjects	91 (84.3%)
Overall, I am satisfied with the flipped classroom approach	94 (87.0%)

## Discussion

The present study shows that the flipped classroom approach is more effective than traditional teaching methods in improving learning outcomes among third-phase MBBS students. Students exposed to the flipped classroom model demonstrated significantly greater post-test score improvement with a large effect size, indicating substantial educational impact. Students also reported positive perceptions regarding engagement, conceptual clarity, and opportunities for self-paced learning.

The flipped classroom demonstrated a large within-group effect, though the between-group effect size was small-to-moderate, indicating a modest but meaningful advantage over traditional teaching. The greater knowledge gain observed with the flipped classroom approach suggests that active, student-centered learning strategies enhance cognitive processing and retention. High levels of student acceptance and satisfaction further support the feasibility of implementing this model in undergraduate medical education.

The findings are consistent with previous Indian studies, reporting improved academic performance, motivation, and engagement following the use of flipped classroom strategies [4,7]. International studies similarly show that flipped classroom models promote higher-order cognitive skills, active participation, and improved examination performance compared to traditional lectures [5,6,9].

By shifting lower-order cognitive tasks such as information acquisition to the pre-class phase, the flipped classroom allows in-class time to be used for higher-order learning activities, including application, analysis, and problem-solving. This approach aligns well with CBME, which emphasizes self-directed learning, active participation, and the development of lifelong learning skills [8]. Sharma et al. (2015) reported that flipped classroom methodology enhances learner participation, promotes critical thinking, and fosters collaborative skills such as communication and teamwork [3]. Similarly, Jawalekar et al. (2024) demonstrated that flipped classroom teaching promotes self-directed learning, a key CBME competency [11], while Jha et al. (2024) further observed that this model facilitates interactive and competency-focused learning consistent with the learner-centred and outcome-based philosophy of CBME [12]. Collectively, these findings suggest that the flipped classroom is not merely a pedagogical modification but a teaching strategy that supports the attainment of CBME objectives in undergraduate medical education.

The simple and practical design of the study makes it feasible for implementation in routine teaching settings without extensive resource requirements. The use of both objective assessment of knowledge gain and subjective evaluation of student perceptions provides a comprehensive understanding of the effectiveness and acceptability of the flipped classroom approach. Additionally, the study contributes region-specific evidence from an Indian undergraduate medical education setting, where data on innovative teaching methods remain limited.

Despite its strengths, the present study has certain limitations. Being conducted at a single institution and within a single discipline may limit the generalizability of the findings. Additionally, the study did not control for variability in topic complexity, which could have influenced student engagement and learning outcomes across sessions. The perception assessment was questionnaire-based and therefore susceptible to response bias. The evaluation focused on short-term knowledge gain, and long-term knowledge retention and impact on clinical performance were not assessed. Broader acceptance and applicability of the flipped classroom approach can be better determined through future studies involving multiple disciplines and institutions. Nevertheless, the study provides evidence that learning has occurred and supports the effectiveness of the flipped classroom as a teaching-learning strategy.

Future research should involve multicentric studies with larger sample sizes and longitudinal designs to evaluate long-term retention and clinical competence. Further exploration of different combinations of pre-class and in-class activities may help optimize flipped classroom design and support its wider integration into undergraduate medical education.

## Conclusions

The flipped classroom approach was found to be more effective than traditional teaching methods in enhancing academic performance, student engagement, and conceptual understanding among third-phase MBBS students. Students demonstrated positive perceptions toward this learner-centered approach. Integration of flipped classroom strategies into routine medical teaching may improve learning outcomes and support the goals of competency-based medical education.

## Appendices

Pre-intervention test questionnaire

Session 1. Topic: Infliction of Injury, Medico-Legal Certificate (MLC), and Weapon Examination

1. Which section of IPC defines culpable homicide? a) 300 b) 307 c) 299 d) 304

2. Loss of tooth following assault is classified as: a) Simple hurt b) Grievous hurt c) Dangerous injury d) Trivial injury

3. Common site for hesitation cuts is: a) Back b) Palms c) Wrist 7. d) Buttock

4. Temporary loss of vision for 10 days following injury is: a) Simple hurt b) Grievous hurt c) Fatal injury d) Non-medicolegal injury

5. An injury which endangers life but does not cause death is termed: a) Fatal injury b) Dangerous injury c) Grievous injury d) Simple injury

6. Causing hurt by dangerous weapon is punishable under: a) 323 IPC b) 324 IPC c) 325 IPC d) 352 IPC

7. Dowry death is punishable under: a) 302 IPC b) 304A IPC c) 304B IPC d) 306 IPC

8. Which is a sign of vitality? a) Dry wound b) Tissue reaction c) Clean margins d) Absence of clot

9. Hurt includes all EXCEPT: a) Bodily pain b) Disease c) Mental shock d) Infirmity

10. The essential distinction between murder and culpable homicide lies in: a) Site of injury b) Weapon used c) Degree of intention d) Number of injuries

Session 2. Topic: Medicolegal Importance (MLI) of Injuries

1. Which among the following is correctly matched? a) Culpable homicide - IPC 300 b) Attempt to murder - IPC 307 c) Murder - IPC 299 d) Abetment of suicide - IPC 306

2. Temporary deafness after assault which improves is classified as: a) Simple hurt b) Grievous hurt c) Dangerous injury d) Fatal injury

3. Typical site for self-inflicted suicidal injuries is: a) Back of trunk b) Abdomen midline c) Wrist d) Gluteal region

4. Fatal injury is: a) Injury sufficient to cause death in ordinary course b) Injury requiring hospitalization c) Injury causing fracture d) Injury causing pain

5. Hurt is defined under IPC section: a) 319 b) 320 c) 323 d) 326

6. Dowry death is punishable under: a) 302 IPC b) 304 IPC c) 304A IPC d) 304B IPC

7. Injury likely to cause death in normal health is: a) Trivial injury b) Dangerous injury c) Fatal injury d) Simple injury

8. Causing hurt with dangerous weapon is punishable under: a) 323 IPC b) 324 IPC c) 325 IPC d) 352 IPC

9. Vitality of wound is indicated by: a) Clean margins b) Absence of bleeding c) Inflammatory reaction d) Dry appearance

10. Hurt does NOT include: a) Bodily pain b) Disease c) Infirmity d) Emotional distress

Post-intervention test questionnaire

Session 1. Topic: Infliction of Injury, MLC, and Weapon Examination

1. Factors affecting wound healing are: a) Infection of the wound b) Nutritional status c) Alcohol consumption d) All three options

2. Infliction of injury over the body depends upon: a) Site of impact b) Weight of the weapon c) Force applied per unit area d) Time of the day

3. In water-containing tissues: a) Impact force is directed to opposite site b) Damage is localized at site of impact c) Damage occurs in all directions d) Damage is less than firm tissues

4. The commonest cause of death in lacerated wound is: a) Fat embolism b) Air embolism c) Infection d) Hemorrhage

5. Injuries can cause death by: a) Embolism b) Malignancy c) Renal failure d) All three options

6. Most important duty of casualty medical officer is: a) Informing the police b) Taking consent c) Treatment d) Taking samples

7. A patient involved in a road traffic accident was initially treated at a Primary Health Centre, where the doctor informed the police and prepared the MLC report before referring the patient to a tertiary care hospital. On receiving the patient, what should the Casualty Medical Officer (CMO) do next?

a) Inform the police again b) Prepare a fresh MLC report c) Inform the police only at discharge d) Make an entry in the MLC register

8. Classification of weapon depends upon all EXCEPT: a) Weight b) Nature of edge c) Length d) Consistency

9. Objective of examination of weapon is: a) Correlate weapon with injury b) Identify assailant c) Identify victim d) All three options

10. Who decides that the case is a medico-legal case? a) Attending doctor b) Police c) Relatives d) Court

Session 2. Topic: MLI of Injuries

1. Which among the following is an incorrect match? a) Murder - IPC 300 b) Abetment of suicide - IPC 304 c) Attempt to murder - IPC 307 d) Culpable homicide - IPC 299

2. A person is slapped and develops temporary hearing loss which is later restored after treatment. The nature of injury should be:

a) Simple b) Grievous c) Dangerous d) Serious

3. Target sites in suicidal injuries are: a) Neck, wrist, left chest b) Exposed parts on same side c) Head, chest, abdomen d) Dorsal portions

4. Bodily injury likely to cause death means: a) The injury is sufficient to cause death in a person of ordinary health. b) Death is the direct and probable consequence of the injury c) The injury is fatal regardless of whether the victim had a pre-existing disease. d) All the above

5. Dangerous weapon is defined under: a) 319 b) 320 c) 324 d) 326

6. Dowry death is charged under: a) 302 b) 304 c) 304A d) 304B

7. Antemortem wound feature EXCEPT: a) Enzyme histochemistry b) Signs of inflammation c) Everted swollen edges d) Non-laminated clot

8. Hurt does NOT include: a) Disease b) Pain c) Infirmity d) Stiffness of joint

9. Not a dangerous injury: a) Cut radial artery b) Broken rib c) Frontal bone fracture d) Thigh laceration

10. Basic difference between murder & culpable homicide: a) Mens rea b) Actus reus c) Knowledge vs intention d) Both intention & knowledge
